# Follow-Up of Neurosarcoidosis With Longitudinally Extensive Myelitis: A Case Report and Review of the Literature

**DOI:** 10.7759/cureus.90238

**Published:** 2025-08-16

**Authors:** Payam Dibaj, Anis A Harun

**Affiliations:** 1 Center for Rare Diseases (ZSEG) Department of Pediatrics, University of Göttingen, University Medical Center (UMG), Göttingen, DEU; 2 Center for Neurology, Asklepios Hospitals Schildautal, Seesen, DEU

**Keywords:** clinical follow-up care, longitudinally extensive transverse myelitis (letm), mri spinal cord, neurosarcoidosis, spinal cord sarcoidosis

## Abstract

A 49-year-old woman presented with a three-month history of neuropathic pain and paresthesia affecting the trunk and all extremities, accompanied by hypesthesia in the upper thoracic region and both thighs. Spinal MRI revealed a longitudinally extensive lesion spanning 12 vertebral segments (C6 to Th10) with dorsal subpial gadolinium enhancement and a characteristic trident sign on axial images. Cerebrospinal fluid (CSF) analysis showed mild lymphocytic pleocytosis and elevated protein and lactate levels. While aquaporin-4 (AQP4) and myelin oligodendrocyte glycoprotein (MOG) antibodies were negative, a significantly elevated concentration of soluble interleukin-2 receptor (sIL-2R) in the CSF (96.1 U/mL; normal <8 U/mL) supported the diagnosis of neurosarcoidosis. Additional findings, including a frontobasal leptomeningeal lesion on brain MRI and enlarged mediastinal lymph nodes, further supported the diagnosis. Treatment with weight-adjusted corticosteroids resulted in rapid clinical improvement and a reduction in MRI enhancement. Four months after treatment initiation, the spinal lesion showed almost complete resolution of gadolinium enhancement and disappearance of the trident sign. Methotrexate was introduced to reduce steroid dependency, resulting in sustained symptom control. Over a four-year follow-up, the patient remained neurologically stable, with no significant MRI findings and normalization of CSF sIL-2R levels. This case highlights the importance of recognizing spinal neurosarcoidosis as a rare but treatable cause of longitudinally extensive transverse myelitis and treating it with appropriate short- and long-term immunosuppressive therapy, especially in patients with significant inflammation. It also highlights the usefulness of MRI and CSF sIL-2R monitoring for diagnosis and long-term treatment.

## Introduction

Neurosarcoidosis is a rare but potentially debilitating manifestation of sarcoidosis, involving the central or peripheral nervous system in approximately 5% to 15% of cases [1]. Spinal cord involvement is particularly uncommon, occurring in less than 1% of sarcoidosis patients, and can present with a wide spectrum of neurological symptoms depending on the affected regions [2,3].

The diagnosis of neurosarcoidosis (with or without spinal cord involvement) remains challenging due to the heterogeneity of clinical presentations and the lack of a single definitive test other than histological examination. Magnetic resonance imaging (MRI) is a key diagnostic tool and often reveals longitudinally extensive lesions of the spinal cord. A recently described radiological feature known as the "trident sign," characterized by a symmetrical, dorsal subpial enhancement pattern on axial spinal MRI, may help distinguish sarcoidosis from other inflammatory myelopathies, including neuromyelitis optica spectrum disorders (NMOSD), myelin oligodendrocyte glycoprotein (MOG) antibody-associated disease (MOGAD), and multiple sclerosis [4].

Cerebrospinal fluid (CSF) analysis frequently shows nonspecific findings, such as mild pleocytosis and elevated protein levels. However, the measurement of soluble interleukin-2 receptor (sIL-2R) in CSF, although nonspecific, has emerged as a potentially useful biomarker for neurosarcoidosis, particularly in the absence of elevated angiotensin-converting enzyme (ACE) or lysozyme levels [5-7]. The presence of mediastinal lymphadenopathy or leptomeningeal enhancement may further support the diagnosis, even in the absence of histological confirmation [6-8].

We present a case of longitudinally extensive myelitis with trident sign and markedly elevated CSF sIL-2R levels, in which a diagnosis of probable neurosarcoidosis was made based on clinical, radiological, and laboratory findings. The patient showed a remarkable clinical and radiologic response to corticosteroids and methotrexate, with sustained remission over a four-year follow-up period.

## Case presentation

A 49-year-old woman presented with a three-month history of neuropathic pain characterized by persistent burning and intermittent neuralgia. The symptoms initially began in both axillae and progressively spread across the trunk. Over time, she developed intermittent paresthesia affecting all four extremities. Neurological examination revealed hypoesthesia in the dermatomes TH1-TH5 bilaterally, as well as in both thighs. Muscle strength testing was normal in all extremities. Deep tendon reflexes were brisk but symmetrical, and there were no pyramidal signs bilaterally. Vibration sense was mildly reduced in the lower extremities, with a bimalleolar score of 3/8. Joint position sense was preserved in all extremities. The Lhermitte sign was negative.

Diagnosis

**Figure 1:**
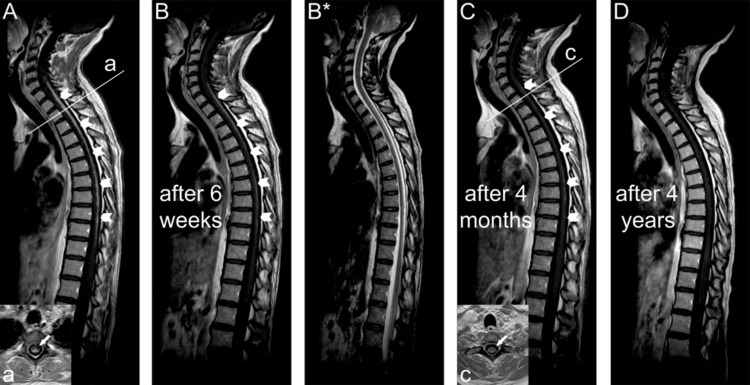
Longitudinal spinal cord lesion with trident sign in neurosarcoidosis before and after treatment Spinal MRI showed dorsal subpial and intramedullary gadolinium enhancement in T1-weighted sequences across 12 vertebral segments from C6 to T10 [A, marked by arrowheads; "a": cross-sectional image at the T2 level with the trident sign (marked by an arrow)]. The subsequent MRI scans showed complete reduction of the spinal lesion within the next four years [B-D, marked by arrowheads in B and C; B* shows the corresponding T2-weighted sequence from the same time point as in B; "c": cross-sectional image at the T1 level, with the trident sign no longer visible (arrow)].

Spinal MRI revealed a longitudinally extensive spinal cord lesion extending from C6 to TH10. T1-weighted sequences with gadolinium contrast demonstrated prominent dorsal subpial and intramedullary enhancement (marked by arrowheads in Figure 1A). Transverse images at the TH2 level exhibited the characteristic "trident sign," a radiological marker suggestive of sarcoid myelitis (marked by an arrow in Figure 1a).

CSF analysis from lumbar puncture revealed mild lymphocytic pleocytosis (27 cells/µL), with elevated protein (1.04 g/L) and lactate (2.9 mmol/L) levels. CSF PCR and serological testing for infectious agents, including herpesviruses, Borrelia, and common bacteria, were negative. Notably, the CSF concentration of sIL-2R was markedly elevated at 96.1 U/mL (normal <8 U/mL), while serum sIL-2R was modestly increased at 755 U/mL (normal <700 U/mL). Neopterin level in CSF was slightly elevated at 1.7 µg/L (normal <1.4 µg/L). ACE and lysozyme levels were within normal limits in both CSF and serum. Testing for aquaporin-4 (AQP4) and MOG antibodies was negative in both serum and CSF, as were antineuronal antibody panels. Quantitative immunoglobulin analysis in CSF and serum showed no overall intrathecal immunoglobulin synthesis; however, the presence of oligoclonal bands exclusively in CSF indicated localized intrathecal IgG production. Erythrocyte sedimentation rate (ESR) and C-reactive protein (CRP) were within the normal range, with a first-hour ESR value below 20 mm. Vitamin status assessment revealed normal levels of vitamin B12, folate, and vitamin B1, with a mildly reduced vitamin B6 concentration.

Additional imaging demonstrated a frontobasal leptomeningeal lesion on brain MRI and mediastinal lymphadenopathy on chest CT. Although histological confirmation was not pursued due to patient refusal, the constellation of clinical findings, spinal and cerebral MRI abnormalities, including the trident sign, and markedly elevated CSF sIL-2R supported a diagnosis of probable neurosarcoidosis, according to established diagnostic criteria [1,6,8].

Treatment and follow-up

The patient was started on weight-adjusted oral corticosteroids, leading to substantial clinical improvement within weeks. Both neuropathic pain and hypoesthesia diminished considerably. Prednisolone tapering was carried out as follows: 1 mg/kg body weight for the first six weeks, followed by monthly reductions of 10 mg until a daily dose of 10 mg was reached, and then, after three months, further reduced to 5 mg daily. Repeat spinal MRI six weeks after therapy initiation revealed significantly reduced gadolinium enhancement (marked by arrowheads in Figure 1B; the corresponding T2-weighted sequence from the same time point is shown in Figure 1B*), and the CSF sIL-2R had decreased to 29.1 U/mL. Four months after treatment initiation, MRI showed near-complete resolution of the spinal lesion (marked by arrowheads in Figure 1C), and the trident sign was no longer visible on axial images (marked by arrow in Figure 1c).

To facilitate corticosteroid tapering and sustain disease remission, methotrexate was introduced. The patient achieved complete resolution of neuropathic pain and paresthesia. Serial annual MRI follow-up over the next four years demonstrated no recurrence of spinal inflammation (Figure 1D), and CSF sIL-2R levels normalized to <8 U/mL one year after initial presentation. Concomitantly, other CSF parameters, including cell count, total protein, lactate, and neopterin, also normalized, whereas oligoclonal bands persisted exclusively in CSF.

## Discussion

Neurosarcoidosis is a rare manifestation of sarcoidosis, accounting for approximately 5%-15% of all sarcoidosis cases, with spinal cord involvement occurring in fewer than 1% of patients [1-3]. Its clinical presentation varies widely, often mimicking other inflammatory, infectious, or neoplastic etiologies, making timely diagnosis particularly challenging. This case illustrates a classic example of longitudinally extensive transverse myelitis (LETM) secondary to probable neurosarcoidosis, successfully managed with immunosuppressive therapy.

Diagnostic challenges and differentials

Spinal neurosarcoidosis can present with longitudinal lesions extending across more than three vertebral segments, a radiological pattern shared with NMOSD, MOGAD, paraneoplastic syndromes, and infectious or vascular etiologies [3]. In this case, both AQP4-IgG and MOG-IgG antibodies were negative, and no infectious or paraneoplastic/antineuronal markers were identified, narrowing the differential diagnosis.

A pivotal diagnostic clue in our patient was the identification of the “trident sign” on axial T1-weighted post-contrast MRI. The trident sign refers to a symmetrical, dorsal subpial enhancement pattern resembling a three-pronged trident, involving both dorsal horns and the central canal region. First described by Flanagan et al. [4], this imaging feature is increasingly recognized as highly suggestive of spinal neurosarcoidosis and is typically absent in NMOSD or MOGAD. Its presence in our case, together with the elevated CSF sIL-2R as well as with the presence of a frontobasal leptomeningeal lesion and mediastinal lymphadenopathy, provided strong support for the diagnosis.

In addition to longitudinal spinal cord lesions, neurosarcoidosis can present with other lesion patterns, such as multinodular enhancements [9]. Nonetheless, the combination of advanced imaging findings with relevant CSF and systemic biomarkers enhances diagnostic accuracy and helps guide timely therapeutic decisions.

Biomarkers and CSF findings

Routine CSF findings in neurosarcoidosis - mild pleocytosis, elevated protein, and normal glucose - are nonspecific [5,6]. However, the measurement of sIL-2R in CSF has recently emerged as a promising biomarker for central nervous system (CNS) sarcoidosis. Several studies have shown that CSF sIL-2R has greater diagnostic sensitivity for neurosarcoidosis than ACE or lysozyme, which were normal in our patient [6,7,10]. The markedly elevated CSF sIL-2R in our case (96.1 U/mL) was consistent with active granulomatous inflammation and normalized with treatment, supporting its utility in both diagnosis and disease monitoring.

Additionally, neopterin, an inflammatory marker of activated macrophages, was slightly elevated in our case. While not specific, this finding further supports the presence of CNS inflammation.

Treatment and clinical implications

Corticosteroids remain the first-line treatment for neurosarcoidosis, often leading to rapid clinical improvement [2,8]. In refractory or steroid-dependent cases, immunosuppressants such as methotrexate, azathioprine, or infliximab may be added [11]. In our patient, the addition of methotrexate enabled tapering of corticosteroids and helped maintain long-term remission. The absence of gadolinium enhancement on MRI during follow-up and the normalization of CSF sIL-2R suggest sustained disease control.

This case underscores the importance of early diagnosis and timely treatment of spinal neurosarcoidosis to prevent irreversible neurological damage. It also highlights the clinical utility of the trident sign on spinal MRI and the measurement of CSF sIL-2R in establishing a diagnosis when histologic confirmation is not feasible.

Clinical significance

Our case contributes to the growing body of evidence supporting the integration of advanced MRI features, such as the “trident sign”, and emerging CSF biomarkers into the diagnostic algorithm for neurosarcoidosis. Given the rarity and potential severity of spinal involvement, increased awareness of these diagnostic clues may reduce diagnostic delays and improve patient outcomes.

According to Nitsch et al. [12], risk factors such as parenchymal CNS lesions and significantly elevated CSF protein levels in our case justify a combined and long-term immunosuppressive strategy to reduce the risk of recurrence. The long-term follow-up in our case underscores the effectiveness of combined immunosuppressive therapy in maintaining remission. This case highlights the importance not only of an accurate diagnosis but also of risk stratification for optimal individualized treatment decisions in neurosarcoidosis.

Study limitations

This case report has a few limitations. First, the diagnosis of neurosarcoidosis was based on clinical, radiological, and laboratory findings without histopathological confirmation, as the patient declined biopsy. Although the diagnostic criteria for probable neurosarcoidosis were met, the lack of tissue confirmation remains a limitation in terms of diagnostic certainty. However, the favorable and sustained clinical outcome following first-line immunosuppressive therapy further supports the diagnosis, as infectious or malignant mimics would be expected to progress under this regimen. Second, this is a single-case report, and the findings, while illustrative, may not be generalizable to all patients with spinal neurosarcoidosis. Finally, although sIL-2R levels in CSF were used to support diagnosis and monitor treatment response, the CSF biomarker remains nonspecific in CNS inflammation, as elevations may also be observed in other conditions such as CNS lymphoma, viral or bacterial meningitis, multiple sclerosis, and other autoimmune or granulomatous disorders affecting the CNS. Despite these limitations, this case offers valuable insights into the diagnostic and therapeutic management of a rare presentation of neurosarcoidosis and contributes to the growing evidence supporting a multimodal diagnostic approach.

## Conclusions

This case highlights the diagnostic and therapeutic challenges of spinal neurosarcoidosis, a rare but treatable cause of LETM. The presence of the trident sign on spinal MRI and markedly elevated sIL-2R levels in the CSF were pivotal in establishing a diagnosis in the absence of histological confirmation. Early initiation of corticosteroids followed by steroid-sparing immunosuppressive therapy with methotrexate led to sustained clinical and radiological remission over a four-year follow-up period. In patients with atypical spinal cord injuries, clinicians should always have a high degree of suspicion for neurosarcoidosis and consider incorporating CSF biomarkers and advanced imaging techniques into the diagnostic workup. This case reinforces the need for timely recognition and long-term management strategies in neurosarcoidosis to prevent irreversible neurological deficits.
